# Monitorization of drug content in furosemide and lorazepam tablets stored in multidose pill boxes

**DOI:** 10.4103/0975-7406.72140

**Published:** 2010

**Authors:** Jessica Martins, Magda Oliveira, Maria Tapiço, Tânia Nascimento, Ana Grenha, Luis Braz

**Affiliations:** 1 School of Health, University of Algarve, Portugal; 2 CBME/IBB, LA - Centre for Molecular and Structural Biomedicine, Institute for Biotechnology and Bioengineering, University of Algarve, Portugal; 3 School of Health, University of Algarve; CIQA - Centre for Research in Chemistry of Algarve, University of Algarve, Portugal

**Keywords:** Furosemide, lorazepam, multidose pill boxes, tablets drug content

## Abstract

**Background:**

Therapeutic nonadherence is a major health problem, particularly when therapeutic regimens are complex and long-lasting. Therefore, tools such as multidose pill boxes have been designed to provide the means for higher therapeutic compliance. However, no studies are available reporting on their capacity to keep the drug content of the stored tablets unaltered.

**Objective:**

This work aimed at monitoring the drug content of tablets stored in multidose boxes for a period of two weeks.

**Materials and Methods:**

Furosemide and lorazepam were selected as model drugs, given their frequent chronic use, which is coherent with the profile of medicines susceptible of storage in the referred boxes. Variations of the tablets drug content were assessed as a function of temperature (25°C and 40°C) and the presence of blister.

**Results and Discussion:**

The obtained results allowed concluding that concerning temperature, only lorazepam tablets registered drug content alterations and only when stored at 40°C. On the other side, it was concluded that the absence of blister does not compromise the drug content of the studied tablets.

**Conclusion:**

In the specific conditions of this study, the storage of these medicines in multidose boxes is considered reliable and adequate.

Worldwide, old age population has increased from less than 100 million persons, before World War II, to 590 millions in 2000. It is estimated that this group shall continue to grow, reaching 1,2 billion in 2025.[1]

Geriatric patients are affected by a great number of chronic diseases, being frequently submitted to treatments based on a high number of medicines, which result many times in problems related with administration errors, adverse reactions, administration oblivious and difficulties in maintaining adequate therapeutic regimens.[2] These issues often lead not only to therapeutic failure, but they also cause an increase in morbidity and mortality, further generating raised healthcare costs. Thus, efforts to enhance therapeutic adherence might represent a great investment in controlling chronic diseases and, in this sense, some tools have recently emerged to the market, such as multidose pill boxes.[3–5] Nevertheless, in spite of their frequent use, and especially considering that many patients use this device to store medications without the blister, ensuring their safety and efficacy demands an evaluation of the stored medicines’ stability, particularly concerning drug content.

Medicines applied in the treatment of chronic diseases, which require repeated and continuous administration, are those more susceptible of storage in the multidose pill boxes. Hypertension[6] and depression[7] are two disorders affecting a high number of patients worldwide. Actually, in Portugal, the prevalence of hypertension was approximately 44% in 2005[8] and an increased use of antihypertensives was observed between 1995 and 2001, the number of dispensed medicine packages rising from 7.6 to 13.4 millions.[9] In turn, anxiolytics, sedatives, and hypnotics, namely benzodiazepines were, in 2004, the most used psycothropic drugs.[10]

The objective of this work was to monitor the drug content of furosemide and lorazepam tablets when stored in multidose pill boxes, as these drugs are frequently used in a chronic manner, thus corresponding to the profile of medicines susceptible of storage in multidose boxes. The referred monitorization was performed as a function of temperature and presence of blister.

## Materials and Methods

### Materials

Conventional tablets of furosemide (40 mg) and lorazepam (2.5 mg) were used to perform these studies. Sodium hydroxide was supplied by Josç M. Vaz Pereira (Portugal) and ethanol 96% (v/v) by Álcool e Gçneros Alimentícios, S.A (Portugal). Distilled water was used throughout.

### Assay conditions

Drug content of furosemide and lorazepam tablets was evaluated during 15 days of storage in multidose pill boxes, considering temperature and the presence of blister as variables. To do so, the tablets’ samples (stored in the multidose boxes) were placed in ovens (Binder FED 115, Germany) regulated to 25°C and 40°C, which correspond, respectively, to room temperature and an extreme temperature. Tablets’ drug content was determined at programmed days. It is important to refer that the condition of 40°C was used in order to extreme conditions and the resultant behavior of the assayed formulations was observed, not corresponding to a temperature frequently registered.

A control corresponding to the tablets’ drug content at time zero tablets not submitted to the study conditions was used for each drug. Furosemide tablets were protected from light during the whole assay due to their photosensitivity.[11,12]

### Determination of drug content

#### Lorazepam

To determine lorazepam drug content, two tablets were weighed (Kern ALS120-4, Germany) and ground. Afterward, the equivalent to 2.5 mg of the drug was weighed and dissolved in 20 mL of 96% ethanol (v/v), with subsequent stirring for 1 hour (magnetic stirrer, VELP Scientifica ARE, Italy). The volume was then completed to 50 mL using the same solvent and this solution was centrifuged for 10 minutes at 5000 rpm (Avanti^™^ Centrifuge J-25I, Beckman Coulter^™^, rotor JA20, USA). Finally, 5 mL of the resultant supernatant were collected and diluted with 96% ethanol (v/v) to a final volume of 50 mL. This assay was performed in triplicate.

Lorazepam drug content was determined in the obtained solution by spectrophotometry (Bausch and Lomb, Milton Roy Spectronic 1201, USA), the absorbance being measured at 230 nm (1150 was used as specific absorbance in this wavelength). Concentration was determined by application of the Lambert-Beer law:

A = εbc,

where A is the measured absorbance, ε is the molar absortivity (L.cm^−1^.mol^−1^); *b* represents the cell path length (cm), and *c* is the concentration of the solution (mol.L^−1^).[13]

The molar absortivity was calculated using the following formulae:

$$A_{1\%1\mathrm{cm}}\;=\;10\varepsilon /M_{r},$$

where A_1%/1cm_ is the specific absorbance, ε is the molar absortivity (L.cm^−1^.mol^−1^). and M_r_ is the molar mass (g/mol).[13]

#### Furosemide

The procedure to determine furosemide drug content involved weighing (Kern ALS120-4, Germany) and grinding of three tablets. Afterward, the equivalent to 80 mg of the drug were weighed and dissolved in 150 mL of sodium hydroxide 0.1 M, being then completed to a volume of 250 mL. After filtering, 2.5 mL of the obtained solution were diluted to a final volume of 100 mL with sodium hydroxide 0.1 M. This assay was performed in triplicate.

The absorbance of this furosemide solution was determined at 271 nm (580 was used as specific absorbance in this wavelength)[13] and drug concentration was calculated according to the procedure described previously for lorazepam.

### Statistical analysis

The t-test was applied to analyze the obtained data. All analyses were run using the Statistical Package for Social Sciences (SPSS) program (version 17) and differences were considered to be significant at a level of *P* < 0.05.

## Results and Discussion

### Lorazepam assay

**Figure 1:**
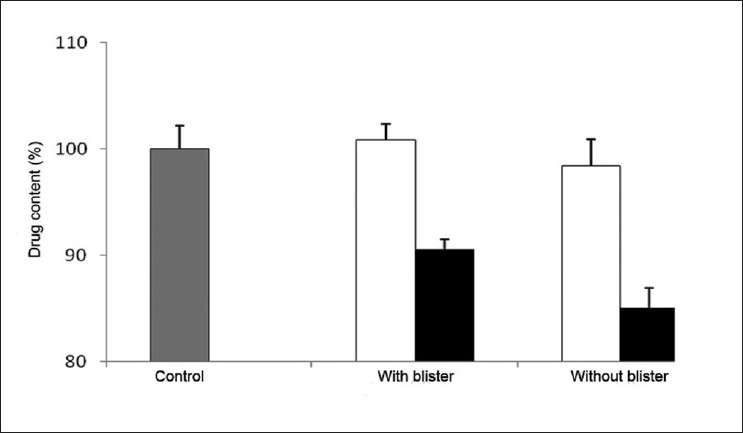
Content of lorazepam determined at day 15 in tablets stored in multidose pill boxes with and without blister at (□) 25°C and (■) 40°C

Upon analyzing the obtained data and comparing the drug content determined after 15 days of incubation of the tablets at 25°C with that of the control tablets not submitted to the study conditions, it was observed that no significant variations occurred, independently of the presence of blister [Figure 1]. In contrast, storing the tablets at 40°C induced strong alterations on the drug content upon the same period of time (*P* < 0.05).

**Figure 2:**
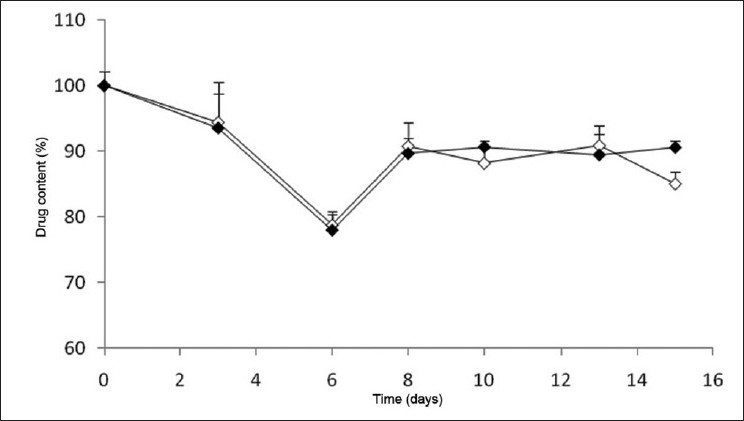
Variation of lorazepam content in tablets stored in multidose pill boxes at 40°C, (◊) without and (♦) with blister

Moreover, as shown in Figure 2, this drug content decrease observed at 40°C has started to be significant, in comparison with the control, from day 6 (*P* < 0.05) for both the tablets stored with and without the blister. As referred in the methodology section, the storage at 40°C is, however, an extreme condition and so, this alteration could be predictable if one considers that drug stability can be affected by light, temperature, pH, reactions with excipients, or even by the storage method.[14] In fact, according to the Portuguese Pharmacopeia, the recommended storage temperature for this drug is 25°C,[13] which reinforces the expectancy of this behavior. This figure also demonstrates that in the last day of the assay performed at the temperature of 40°C, the tablets stored with blister evidence higher drug content as compared to those stored in its absence (*P* < 0.05), this difference not being observed in the previous days. This could suggest that in extreme temperature conditions and in periods as long as 15 days, the blister could confer some kind of protection to the tablet. This fact could only be confirmed if the study was performed for a longer period of time (> 15 days), which has not a particular interest for the subject, as patients are not expected to store medicines in multidose pill boxes for more than 2 weeks.

**Figure 3:**
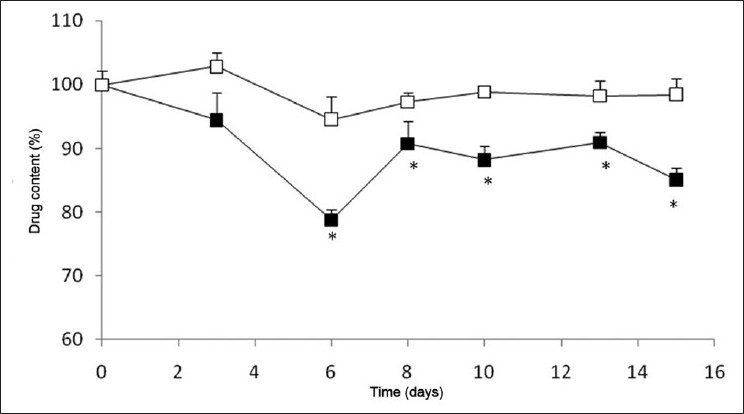
Variation of lorazepam content in tablets stored without blister in multidose pill boxes at (□) 25°C and (■) 40°C (*P < 0.05)

Figure 3 displays the drug content variation in lorazepam tablets stored without blister in multidose pill boxes, at both the assayed temperatures. The results have shown that from the 6th day of the assay, the drug content determined in tablets stored at 40°C is lower than that of those stored at 25°C (*P* < 0.05). Tablets stored with blister have registered similar results (data not shown). These results reveal a temperature-dependent behavior for this specific drug, reinforcing the need to store these tablets at 25°C, as mentioned above and as advised in the Portuguese Pharmacopeia.

In this manner, considering the presented results, it was demonstrated that the storage of lorazepam tablets inside multidose boxes that remain under room temperature is efficient, but extreme temperatures affect their stability. Moreover, the storage of the tablets in the presence of blister has been found to not confer additional protection.

### Furosemide assay

**Figure 4:**
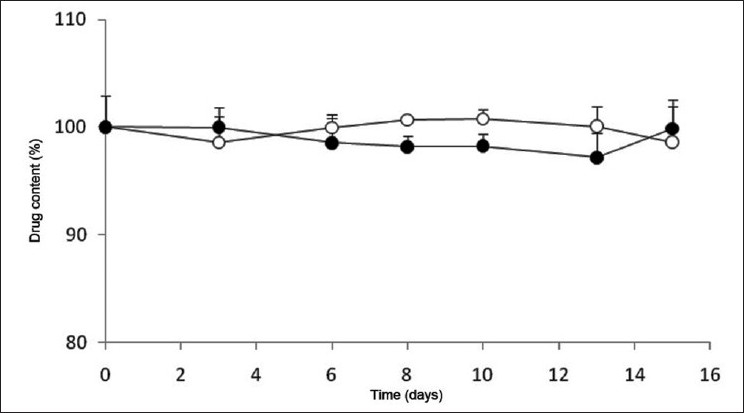
Variation of furosemide content in tablets stored without blister in multidose pill boxes at (○) 25°C and (●) 40°C

Results obtained with furosemide tablets were not coincident with those described above for lorazepam. Actually, the determined data indicated that comparing with the control, the drug content of furosemide tablets stored under the temperatures of 25°C and 40°C without blister remained stable during the 15 days of the study [Figure 4]. As expected, the same occurred with the tablets stored in the presence of blister, which displayed similar results (data not shown).

**Figure 5:**
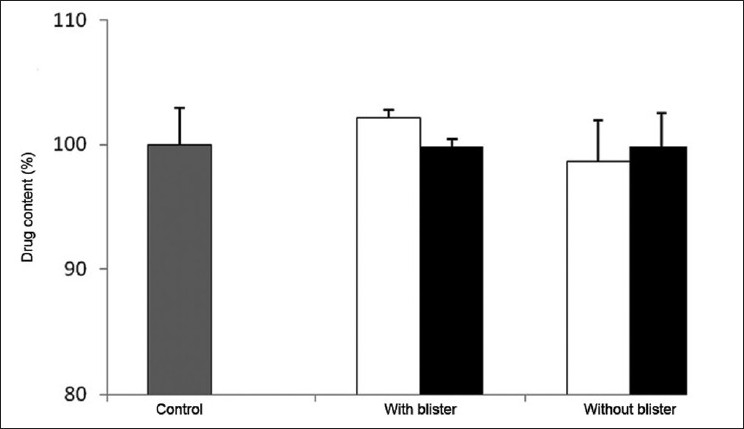
Content of furosemide determined at day 15 in tablets stored in multidose pill boxes with and without blister at (□) 25°C and (■) 40°C

It is important to highlight that even at the end of the 15 days that the assay lasted, in contrast to what was observed for lorazepam, none of the assayed variables affected the furosemide content of the tablets, as shown in Figure 5.

The general analysis of these results allowed to conclude that neither the effect of temperature, even when extreme, nor the presence of blister compromise the stability of furosemide tablets, which are stored in multidose pill boxes, thus rendering this an efficient tool to store these tablets for periods up to 2 weeks.

## Conclusion

Taking into account the determination of the drug content evolution in the studied furosemide and lorazepam tablets and considering the specific conditions of this assay, it could be concluded that the use of multidose pill boxes is efficient and that the ideal temperature to store tablets is 25°C, especially in the case of lorazepam. In view of the different behaviors displayed by the analyzed formulations, as well as the vast number of medicines susceptible of storage in multidose pill boxes, it would be of great interest to extend this study to a larger number of formulations, in order to establish a more conclusive observation on the efficacy of this tool.
